# Fracture Forces of Dentin after Surface Treatment with High Speed Drill Compared to Er:YAG and Er,Cr:YSGG Laser Irradiation

**DOI:** 10.1155/2016/8517947

**Published:** 2016-01-24

**Authors:** Rene Franzen, Nasrin Kianimanesh, Rudolf Marx, Asma Ahmed, Norbert Gutknecht

**Affiliations:** ^1^Department of Conservative Dentistry, Periodontology and Preventive Dentistry, RWTH Aachen University Hospital, Pauwelsstrasse 30, 52074 Aachen, Germany; ^2^AALZ Aachen Dental Laser Center, Center for Biomedical Technology, RWTH Aachen Campus, Pauwelsstrasse 17, 52074 Aachen, Germany; ^3^Medical Material Science, RWTH Aachen University Hospital, Pauwelsstrasse 30, 52074 Aachen, Germany

## Abstract

Dental tooth restorative procedures may weaken the structural integrity of the tooth, with the possibility of leading to fracture. In this study we present findings of coronal dentin strength after different techniques of surface modification. The fracture strength of dentin beams after superficial material removal with a fine diamond bur high speed drill hand piece, Er:YAG (2.94 *μ*m, 8 J/cm^2^), and Er,Cr:YSGG (2.78 *μ*m, 7.8 J/cm^2^) laser irradiation slightly above the ablation threshold was measured by a four-point bending apparatus. Untreated dentin beams served as a control. A total of 58 dentin beams were manufactured from sterilized human extracted molars using the coronal part of the available dentin. Mean values of fracture strength were calculated as 82.0 ± 27.3 MPa for the control group (*n* = 10), 104.5 ± 26.3 MPa for high speed drill treatment (*n* = 10), 96.1 ± 28.1 MPa for Er,Cr:YSGG laser irradiation (*n* = 20), and 89.1 ± 36.3 MPa for Er:YAG laser irradiation (*n* = 18). Independent Student's *t*-tests showed no significant difference between each two groups (*p* > 0.05). Within the parameter settings and the limits of the experimental setup used in this study, both lasers systems as well as the high speed drill do not significantly weaken coronal dentin after surface treatment.

## 1. Introduction

Factors related to tooth fracture have been investigated in different studies [1–3]. It is one of the most dramatic clinical situations which might be of concern to both dentists and patients, because it may consequently end up in loss of tooth structure. Therefore, the structural integrity of teeth under stress and how it may be jeopardized by different types of cracks is a very important issue [4]. The maximum stress by itself does not contribute to crack growth but the process of fatigue cycling results in producing cracks on the tooth surface [5]. The fatigue crack growth in human dentin is dependent on variables such as age, tubule orientation and density, and depth below the dentin-enamel junction [6–8].

Understanding the mechanical behavior of dentin under different conditions is crucial. Nevertheless, tooth fracture is multifactorial and restored teeth are more subject to experience cracks and damage during cutting and cavity preparation. Since cracking of teeth can have serious clinical consequences, the technique of cutting has a significant influence on the mechanical properties of the tooth by introducing and initiating cracks during cutting [1, 5, 9–11].

Though cracks of depths up to 71 *μ*m were observed after diamond preparation in enamel, Banerjee et al. (2000) did not find cracks in dentin resulting from the use of burs but reported that sono-abrasion and Carisolv gels “caused flaws” [1, 3]. Yan et al. (2009) on the other hand did not observe flaws within dentin after bur treatment but nevertheless stated that material removal may be related to the cause of fracture [12]. Majd et al. (2012) evaluated the influence of 6-flute tungsten carbide bur and abrasive air-jet with 50 *μ*m abrasive particles for cavity preparations on the mechanical behavior of coronal dentin. The results were compared with the strength of intact control beams. Both methods significantly decreased the fatigue strength of dentin. In the same study, for the bur treatment an overall degradation in the endurance limit of nearly 40% was reported together with an accompanying decrease in fatigue life. It was considered a “critical issue” since this may hinder the dentin to provide a sound foundation for restorative materials [13]. When mechanical instrumentation is used, friction generates heat and hence elevated temperatures, which may cause irreversible damage to the tooth, while the tooth surface shows signs of thermal and mechanical damage in conjunction with a mechanically created smear layer that is formed as a consequence of this technique [14].

Current lasers for hard tissue preparation have been investigated for their abilities to ablate human enamel, dentin, and bone. Among the current commercially available laser systems, the radiation of the erbium lasers (Er:YAG 2.94 *μ*m and Er,Cr:YSGG 2.78 *μ*m) is strongly absorbed in water and mineral components and therefore offers the possibility for removal of hard tissues, caries removal, and cavity preparation with minimally invasive concept and more patient comfort. These lasers allow ablation of hard dental tissues without causing injury to the pulp or significant thermal side effects such as cracking, melting, or charring of the adjacent tissues at rates comparable with high speed dental drills and with less pain and vibration, as long as they are used with correct laser parameters and water sprays [15–20].

In a study by Maung et al. only 2 of the 15 samples ablated with the laser showed the formation of small cracks while 9 out of 15 samples exhibited crack formation with the dental hand piece [21]. Sehy and Drummond (2004) prepared class I or class II MOD cavities in molars using either a high speed hand piece with coarse diamond bur or an Er:YAG laser. The preparation was followed by placement of a resin composite, bulk curing to maximize interfacial stresses, and evaluation of the tooth-composite interface by scanning electron microscopy was performed. Neither method of preparation resulted in consistent or significant evidence of microcracking in dentin [22].

Staninec et al. compared a free-running pulsed Er:YAG laser (pulse duration 135 *μ*s) with a q-switched Er,Cr:YSGG laser (pulse duration 0.5 *μ*s). The 135 *μ*s pulsed did not create any visible cracks on the irradiated surfaces while the q-switched systems with a 270 times shorter pulse duration, which was additionally operated without an air/water spray, created significant surface cracks from which fractures formed under bending of the specimens [23], pointing out the importance of pulse duration (nanosecond versus microsecond domain) and sufficient water sprays.

The present study addresses the question whether erbium laser surface treatment with fluencies close to the ablation threshold as found for a stopping ablation front in a free-running mode (microsecond domain) may cause weakening of dentin.

## 2. Materials and Methods

Caries-free extracted human molars were collected and completely cleaned of calculus and debris and then sterilized by *γ*-irradiation (Co^60^) minimum absorbed dose of 29 KG. The teeth had been extracted for orthodontic reasons by cooperating dental offices and were donated to science.

The teeth were embedded in resin (Technovit 4000/4002: Heraeus-Kulzer, Wehrheim, Germany) and sectioned in buccolingual direction and then in mesiodistal direction to prepare dentin beams approximately 1.5 mm × 1.5 mm × 9 mm. The beams were roughly shaped with 400 grit and finished with 800 grit. The procedure of preparing dentin beams was adopted from the description of Staninec et al. [23]. The final dimensions after polishing are listed in Tables 1 [Table tab2] [Table tab3]–4. The buccal surface was marked for later orientation of the specimen. The teeth and beam samples were stored in distilled water throughout the experiment with 0.9% NaCl.

The beams were assigned to two laser groups each containing 20 samples (groups 3 and 4) and two control groups each containing 10 samples (groups 1 and 2). In the control groups beams were kept either untreated (group 1), serving as a negative control, or treated with a fine diamond bur in a high speed hand piece under water cooling (group 2), serving as a positive control.

In the laser groups 3 and 4 beams were irradiated by either a free-running Er:YAG laser system 2.94 *μ*m (Lightwalker system, Fotona d.d., Slovenia) or a free-running Er,Cr:YSGG 2.79 *μ*m (iPlus system, Biolase Inc., Irvine, CA, USA) both with a total radiation exposure of 10 seconds while their laser beams were scanned along the whole length of the dentin beams with one back and forth motion in mesiodistal direction and vice versa.

Parameters used in the groups were as follows:

- Group 1: untreated control (negative control).
- Group 2: fine diamond bur in a high speed hand piece with water cooling (positive control).
- Group 3: Er,Cr:YSGG used with a “*Gold*” type hand piece, MZ6 glass tip (diameter 600 *μ*m), pulse duration 60 *μ*s, 25 mJ, 10 Hz, water 80%, air 50% (adjustable spray), and fluence 8 J/cm^2^ on the specimen's surface.
- Group 4: Er:YAG laser used with an “*H14*” hand piece, glass tip (diameter 800 *μ*m), pulse duration 100 *μ*s, 40 mJ, 10 Hz, water 60%, air 40% (adjustable spray), and fluence 7.8 J/cm^2^ on the specimen's surface.

Note that different pulse energies were chosen in groups 2 and 3 to compensate for different glass tip diameters of the laser systems in order to achieve a similar fluence. Around 4 J/cm^2^ has been reported for the ablation threshold of dentin for Er:YAG laser irradiation [23, 24].

Laser parameters for groups 3 and 4 were chosen in such a way that the treated surface of the dentin beams was exposed to fluencies similar to those at the bottom of laser created cavities. While such cavities are obviously being created with significantly higher fluencies (in the order of 60 J/cm^2^ for typical clinically used energies of 300 mJ and a beam cross section of 800 *μ*m), the point where the ablation front stops moving into the dentin, hence the ablative process stopping, is where the newly created cavity floor is found. Therefore, the decision to use fluencies slightly above the ablation thresholds of 7-8 J/cm^2^ was chosen to allow a slight ablative material removal with the ablation front stopping just inside the specimens. Only very little material is expected to be removed from the specimens themselves as due to their dimension for the fracture tests, they would be damaged or broken by the laser pulses if higher fluencies or removal rates are used. Instead, a dentin surface such as being found at a cavity floor is being simulated. Additionally, the dentin beam is irradiated on one surface side only, similar to that in a clinical dentin laser preparation, and the dentin is irradiated from one side.

In a similar fashion, the use of the diamond bur in the control group was moved manually along the length of the dentin beam, allowing it to remove material close to the surface and hence again simulating the floor of a bur-prepared excavation. Note that no EDTA or any similar agent was used to remove the created smear layer. Additionally, we point out that within the limits of our study design we cannot factor in additional sources of stress that could be found in a clinical situation such as forceful movement of the bur creating microfractures or high pulse energies for the laser systems creating similar incidences; however, to our knowledge the latter incidences have not been reported in the literature for free-running pulsed lasers.

Treated surfaces were oriented perpendicularly to the marked buccal surface; hence the direction of dentinal tubules was perpendicular to the treated surface and parallel to the direction of force of the bending test. That would be the same direction as the functional force naturally occurs. Each dentin beam was placed in a mechanical testing machine in a four-point bending apparatus (Zwick/Roell Z5.0, Zwick GmbH, Ulm, Germany) and tested with increasing load at a displacement rate of 1 mm/min until failure. The bending strength *B* can be calculated as shown in what follows:

(1) $$\begin{array}{c} B=\frac{3\left(l-c\right)F}{2bh^{2}} \end{array}$$

using the following variables:  *l* = lower distance of loading points = 7.2 mm,  *c* = upper distance of loading points = 1.8 mm,  *b* = width of the samples,  *h* = thickness of the samples, and  *F* = breaking force.

Each beam was positioned in a four-point bending apparatus with 1.8 mm distance to the upper loading points and 7.2 mm to the lower loading points until fracture occurred under the load. Load at fracture recorded as breaking force *F* and bending strength *B* of each beam was calculated accordingly.

## 3. Results

Final dimensions of the dentinal beams after preparation, breaking force, and bending strength are shown in Tables 1–4.

**Figure 1:**
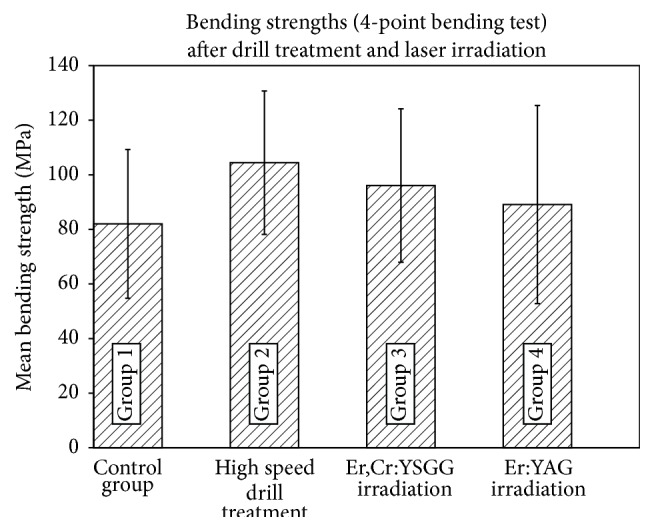
Illustration of the 4-point bending test results showing bending strengths for the control and the 3 test groups.

Mean values of bending strength were calculated as 82.0 ± 27.3 MPa for the unprepared control group, 96.1 ± 28.1 MPa for Er,Cr:YSGG laser irradiation, 89.1 ± 36.3 MPa for Er:YAG laser irradiation, and 104.5 ± 26.3 MPa for high speed drill conditioned surfaces. These values are presented in Figure 1. Independent *t*-tests showed that there were no significant differences between each two groups (*p* > 0.05).

## 4. Discussion

The effect of erbium family lasers on tooth structure during cavity preparation has been investigated in several studies. Various pulse durations and repetition rates and different energy and power parameters were investigated in these studies regarding the micromorphological aspect of enamel and dentin, ablation speed, depth, and/or volume [15, 25–31].

On the other hand, bur tooth preparation is associated with metallic noise and vibration that might cause discomfort and anxiety of the patient, as well as cracks and tooth weakening [1, 13, 21]. Less pain, noise, and vibration have been reported with laser cavity preparation [17, 32]. Bactericidal and anti-infective effects are the other aspects that could be expected [33].

Staninec et al. described a difference regarding fracture under bending between a free-running Er:YAG laser of 135 *μ*s and a q-switched Er,Cr:YSGG laser of 0.5 *μ*s pulse duration. While the Er:YAG treated surfaces did not show visible cracks, the Er,Cr:YSGG treated surfaces showed significant surface cracks. They reported that this resulted in significant weakening for the Er,Cr:YSGG treated specimens. This was explained by the q-switch laser generating mechanical and thermal shock waves, thermal expansion, and recoiling ablation debris [23]. It is to be noted that in addition to the drastically reduced pulse duration (135 versus 0.5 *μ*s) the irradiation with the Er,Cr:YSGG laser was also performed without a water spray which represents an experimental setup which does not simulate a clinical situation.

Therefore, in the presented study, the pulse durations meet clinical requirements for dentin preparation with pulse durations of 50–100 *μ*s typically found on free-running systems (flash-lamp operated). It is noteworthy that q-switched erbium lasers are so far not used clinically in dentistry and that it is well known that water sprays must be used during hard tissue preparations.

In our study, fracture strengths of dentinal beams irradiated with Er:YAG and Er,Cr:YSGG laser were compared with bur preparation and untreated intact beams in a 4-point bending test. There were no significant differences among groups. 4 J/cm^2^ for Er:YAG laser irradiation has already been reported as the ablation threshold for dentin preparation [23, 24]. The fluencies above the ablation threshold for dentin preparation were selected in order to simulate a cavity floor on the surface of the dentin beams within the limits of our experimental setup. With the settings used in the present investigation laser irradiation did not weaken the dentinal beams in comparison with intact beams or bur treated beams.

The Er:YAG laser was compared by Sehy and Drummond with coarse diamond bur for preparation of class I and II MOD cavities that were restored by bulk curing composite restoration and no visible evidence of microcracking was found [22].

However in a study of fatigue crack growth rates by Nalla et al. in human dentin, it was concluded that, under simulated physiologic conditions, small flaws in teeth, in the order of 250 *μ*m, will not radically affect their structural integrity as the predicted fatigue lifetime will exceed that of the patient [34]. For this reason we included group 2 (fine diamond bur in a high speed hand piece with water cooling) as positive control to include the possible effect of structural weakening under mechanical treatment of the surface. Another study by Bosa et al. utilizing a q-switched Er:YSGG laser reported on minimal thermal damage to the specimens while observing mechanical damage at higher fluencies [35].

Near infrared imaging of enamel samples irradiated by an industrial marking laser, operating at a wavelength of 9.3 *μ*m with a repetition rate of 300 Hz, compared the peripheral thermal and mechanical damage produced by a standard dental hand piece with a high speed air turbine with the laser. Here 2 out of 15 (13.3%) irradiated samples were reported to have small cracks next to the ablation craters [21]. Without a water spray, mechanical as well as thermal damage occurred. Compared to this, 9 of 15 (60%) samples prepared with a high speed bur exhibited mechanical damage in the form of cracking and evidence of thermal damage [21].

In the present study, constant water spray was used during the whole laser irradiation process for both Er:YAG and Er,Cr:YSGG lasers, as it is also used in clinical treatments of enamel and dentin, either for cavity preparation or for other surface modifications. The importance of continuous spray application is also confirmed by Darling et al. who observed “very clean without large cracks” ablation craters and no accompanying thermal side effects [19]. The water spray acts as a mediator for efficient ablation and minimizes the risk of adverse thermal effects in the pulp tissue. The excessive increase of intrapulpal temperature and the possible thermal damage to the hard dental tissues have restricted Er:YAG laser ablation on dry teeth. Water sprays are, therefore, essential to reduce the side effects of temperature rise on biological tissues during clinical applications of Er:YAG lasers [36–39]. It has been shown that water cooling is essential to avoid destructive temperature increase whether an erbium laser or a high speed hand piece is used for cavity preparation [40]. The water spray is used to clean the irradiated surface, supply a cooling effect, and assist the ablation process [41, 42]. Additionally, the use of Er:YAG lasers without water spray has been reported to cause formation of non-apatite calcium phosphate phases which may be prone to acid dissolution and demineralization and may eventually lead to insufficient bonding of restorations [43].

In a study by Arola and Rouland the rate of fatigue crack growth was evaluated in terms of the dentin tubule orientation and tubule density. They concluded that fatigue crack growth in dentin is dependent on the tubule orientation [7]. For this reason the treated surface of dentin beams in our study was marked in such a way that tubules were perpendicular to the treated surface and in the same orientation for all samples. During the bending test the treated surfaces of the samples were positioned on the tensile side so the load is applied in the same direction in which it occurs naturally.

However, it is important to note that the presented study is to be interpreted with its limitations in mind. We investigated the influence of treatments and irradiations which are expected to influence the material close to the surface for the reason of simulating a standardized cavity floor. However, this situation does not include all effects that may be present in a clinical cavity preparation. For instance, effects such as shock or pressure waves created with higher fluencies and pulse powers are not present in our model. For flash-lamp pumped erbium lasers these shock waves are unlikely to have an effect, if present at all, as was described by Hibst and Keller [44]. Shock waves are more of a concern when pulse durations drop below 1 *μ*s as is the case when using q-switched lasers [23]. Another effect not modeled in our in vitro situation is the act of mechanical drilling into the tooth during an actual cavity preparation; however, this may be partly compensated with the mechanical stress the teeth had experienced when being sawed apart to manufacture the dentin beam specimens from them. Additionally, the thermal influence of drilling into a tooth with clinical parameters has to be taken into account. While thermal weakening of the substance may in principle be possible, the appropriate use of the water sprays of free-running erbium lasers prevents a thermal increase, as was confirmed experimentally by Rizoiu et al. for the case of the Er,Cr:YSGG type system, where even a decrease in temperature of approx. 2°C was observed [45].

## 5. Conclusion

Within the conditions and limitations used in this study no statistically significant difference could be observed in the fracture strength of dentin beams when treating them either with Er:YAG and Er,Cr:YSGG laser irradiation or mechanically by a fine diamond bur in a high speed hand piece. Additionally, no statistically significant difference could be observed between treated and untreated specimens.

## Figures and Tables

**Table 1:** Exact dimensions, breaking force, and bending strength of the untreated control (group 1). The mean value of the bending strength is 82.02 MPa with a standard deviation of 27.25 MPa.

Number	Thickness/mm	Width/mm	Breaking force/N	Bending strength/MPa
1	1.20	1.90	37.90	112.20
2	1.96	1.60	84.00	110.70
3	1.36	1.90	32.00	73.76
4	1.44	1.88	6.58	13.67
5	1.77	1.66	55.30	86.13
6	1.58	1.86	45.20	78.85
7	1.37	1.85	37.20	86.78
8	1.40	1.86	38.00	84.43
9	1.30	1.75	34.40	94.21
10	1.88	1.77	61.40	79.50

**Table 2 tab2:** Exact dimensions, breaking force, and bending strength of the Er,Cr:YSGG irradiated specimens (group 2). The mean value of the bending strength is 96.08 MPa with a standard deviation of 28.08 MPa.

Number	Thickness/mm	Width/mm	Breaking force/N	Bending strength/MPa
1	1.49	1.90	35.70	68.55
2	1.65	1.80	59.80	98.84
3	1.54	1.87	67.70	123.65
4	1.83	1.46	89.10	147.61
5	1.16	1.88	21.20	67.88
6	1.64	1.95	72.10	111.35
7	1.27	1.75	37.30	107.04
8	1.38	1.89	33.10	74.49
9	1.50	1.83	61.20	120.39
10	1.20	1.80	18.00	56.25
11	1.44	1.74	27.40	61.51
12	0.93	1.70	20.30	111.83
13	1.07	1.86	27.90	106.12
14	1.14	1.87	21.20	70.66
15	1.68	1.88	79.30	121.05
16	1.55	1.73	76.50	149.09
17	1.14	1.90	24.90	81.68
18	1.13	2.37	25.50	68.25
19	1.98	1.00	53.70	110.95
20	1.65	1.80	48.20	79.67

**Table 3 tab3:** Exact dimensions, breaking force, and bending strength of the Er:YAG irradiated specimens (group 2). The mean value of the bending strength is 89.12 MPa with a standard deviation of 36.32 MPa.

Number	Thickness/mm	Width/mm	Breaking force/N	Bending strength/MPa
1	1.27	1.76	33.00	94.16
2	1.02	1.86	36.00	150.69
3	1.24	1.88	25.90	72.57
4	2.00	1.22	70.50	117.02
5	1.86	1.22	30.70	58.92
6	1.57	1.70	60.00	115.98
7	1.45	1.92	60.10	120.59
8	1.64	1.85	38.60	62.84
9	1.26	1.90	27.70	74.38
10	1.75	1.84	43.00	61.81
11	1.59	1.50	62.40	133.29
12	1.14	1.60	18.40	71.68
13	1.75	1.57	31.50	53.07
14	1.97	1.80	90.60	105.05
15	1.94	1.04	29.00	60.01
16	1.98	1.65	118.00	147.76
17	1.81	1.80	63.10	86.67
18	2.00	1.42	38.90	55.47

**Table 4:** Exact dimensions, breaking force, and bending strength of the specimens treated by fine diamond bur in a high speed hand piece (group 1). The mean value of the bending strength is 104.47 MPa with a standard deviation of 26.31 MPa.

Number	Thickness/mm	Width/mm	Breaking force/N	Bending strength/MPa
1	1.15	2.12	39.90	115.27
2	1.75	1.05	34.20	86.15
3	1.12	1.89	24.40	83.36
4	1.55	1.81	44.10	82.15
5	1.56	1.67	49.10	97.86
6	1.35	1.79	48.20	119.68
7	1.40	1.79	33.60	77.57
8	1.65	1.87	55.90	88.94
9	2.00	1.09	78.70	146.21
10	1.57	1.86	83.50	147.52
